# *APOE* Variants in an Iberian Alzheimer Cohort Detected through an Optimized Sanger Sequencing Protocol

**DOI:** 10.3390/genes12010004

**Published:** 2020-12-22

**Authors:** Ricardo D. González, Iva Gomes, Catarina Gomes, Rita Rocha, Luís Durães, Patrícia Sousa, Manuel Figueruelo, Maria Rodríguez, Carmen Pita, Roberto Hornero, Carlos Gómez, Alexandra M. Lopes, Nádia Pinto, Sandra Martins

**Affiliations:** 1 IPATIMUP–Institute of Molecular Pathology and Immunology of the University of Porto, 4200-135 Porto, Portugal; ricardod.gonzalezc@gmail.com (R.D.G.); igomes@ipatimup.pt (I.G.); catarinagomesbq@gmail.com (C.G.); rrocha@ipatimup.pt (R.R.); alopes@ipatimup.pt (A.M.L.); smartins@ipatimup.pt (S.M.); 2 i3S–Instituto de Investigação e Inovação em Saúde, Universidade do Porto, 4200-135 Porto, Portugal; 3 Associação Portuguesa de Familiares e Amigos de Doentes de Alzheimer, 4455-301 Lavra, Portugal; luis.duraes@alzheimerportugal.org (L.D.); patricia.sousa@alzheimerportugal.org (P.S.); 4 Asociación de Familiares y Amigos de Enfermos de Alzheimer y Otras Demencias de Zamora, 49021 Zamora, Spain; direccion@alzheimerzamora.com (M.F.); psicologia@alzheimerzamora.com (M.R.); carmenpitag@gmail.com (C.P.); 5 Grupo de Ingeniería Biomédica, Universidad de Valladolid, 47011 Valladolid, Spain; robhor@tel.uva.es (R.H.); carlos.gomez@tel.uva.es (C.G.); 6 Centro de Investigación Biomédica en Red en Bioingeniería, Biomateriales y Nanomedicina (CIBER-BBN), 47011 Valladolid, Spain; 7 Instituto de Investigación en Matemáticas (IMUVA), Universidad de Valladolid, 47011 Valladolid, Spain; 8 Centro de Matemática da Universidade do Porto, 4169-007 Porto, Portugal

**Keywords:** late onset Alzheimer’s disease (LOAD), *Apolipoprotein E* (*APOE*), rs429358, rs7412, sanger sequencing, disease risk

## Abstract

The primary genetic risk factor for late onset Alzheimer’s disease (LOAD) is the *APOE4* allele of *Apolipoprotein E* (*APOE*) gene. The three most common variants of *APOE* are determined by single nucleotide polymorphisms (SNPs) rs429358 and rs7412. Our aim was to estimate allele and genotype frequencies of *APOE* variants in an Iberian cohort, thus helping to understand differences in *APOE*-related LOAD risk observed across populations. We analyzed saliva or buccal swab samples from 229 LOAD patients and 89 healthy elderly controls (≥68 years old) from Northern Portugal and Castile and León region, Spain. The genotyping was performed by Sanger sequencing, optimized to overcome GC content drawbacks. Results obtained in our Iberian LOAD and control cohorts are in line with previous large meta-analyses on *APOE* frequencies in Caucasian populations; however, we found differences in allele frequencies between our Portuguese and Spanish subgroups of AD patients. Moreover, when comparing studies from Iberian and other Caucasian cohorts, differences in *APOE2* and *APOE4* frequencies and subsequent different *APOE*-related LOAD risks must be clarified. These results show the importance of studying genetic variation at the *APOE* gene in different populations (including analyses at a regional level) to increase our knowledge about its clinical significance.

## 1. Introduction

Alzheimer’s disease (AD) is a progressive neurodegenerative disorder associated with cognitive decline and the leading cause of progressive dementia (60–80% cases) [1]. It is one of the most severe brain disorders of elderly and a major public health problem due to the increased life expectancy observed in most populations [2,3]. It is estimated that by 2030 around 74.7 million people worldwide may be affected with the pathology [4], being noteworthy that AD is highly disruptive also for families and caregivers. Due to its continuum profile, different stages (mild, moderate, and severe) can be observed throughout AD evolution depending on the level of functionality of the patient. Prior to AD, individuals usually develop mild cognitive impairment, a condition that does not necessarily progress to this dementia [5].

The definitive AD diagnosis is only possible after microscopic examination of the patient’s brain, by observing amyloid-β (Aβ) and neurofibrillary tangles formation and accumulation [6]. As this is solely possible with a *post mortem* exam, psychological and molecular tests are used to evaluate memory and thinking skills, as well as expression patterns of genes and proteins, therefore assessing a preliminary diagnosis of the disease [7]. Further, the search for reliable peripheral biomarkers, in easily accessible biological sources as urine, blood and saliva, is of utmost importance. This will open the door for an early diagnostic evaluation of patients, but also to identify individuals at risk, thus allowing the initiation of pharmacological and non-pharmacological therapies to treat symptoms more promptly, which has been proven to be more effective [8,9,10].

Despite its multifactorial profile, the primary genetic risk factor for late onset Alzheimer’s disease (LOAD) is the *Apolipoprotein E* (*APOE*) gene [11]. *APOE* encodes a 34-kDa 299 amino acid protein that plays a key role in the transport and metabolism of plasma cholesterol and triglycerides, in addition to injury repair in the brain [11]. The three major isoforms differ in residues 112 and 158 (APOE2: Cys^112^, Cys^158^; APOE3: Cys^112^, Arg^158^; APOE4: Arg^112^, Arg^158^). These changes affect the protein structure and influence its capability to bind to lipids and receptors, as well as to regulate Aβ production, aggregation, and clearance, which differently affects the risk of developing AD [12]. Mechanistically, Aβ-independent pathways also link the *APOE* status with AD risk, namely, (i) in the process of delivering cholesterol and essential lipids for the maintenance of synaptic integrity and plasticity, (ii) in the regulation of the immune system, and (iii) in maintaining vascular health (reviewed in [3]). Individuals expressing the *APOE4* allele display a higher risk of developing AD, in a dose-dependent fashion: one copy of this allele increases the risk up to 3–4 times, while two copies will enhance it to 8–12 times [13]. On the other hand, the *APOE2* allele confers a reduced risk of AD, with cognitively normal *APOE2* carriers showing a lower rate of hippocampal atrophy, higher cerebrospinal fluid Aβ, and lower phospho-Tau protein expression when compared to individuals with the *APOE3/APOE3* genotype [14].

Frequency of *APOE* variants varies considerably worldwide, previously shown to be population and geographically-associated [15]. Therefore, screening *APOE* in several populations is of great importance to assess ethnicity-specific differences in the risk for AD and other pathologies influenced by the *APOE* genotype, such as stroke, cardiovascular diseases, and other neurodegenerative disorders [16,17]. In AD, given the preponderant role of *APOE* in developing the disease, this screening can facilitate its diagnosis by helping the identification of individuals at higher risk, thus contributing to an earlier intervention to delay AD symptoms. When sampling from different geographical origins, one may also attain a more complete picture of the spectrum of genetic variation of *APOE* in order to better understand differences of AD risk at a regional level.

In the present study, we aimed to estimate the frequency of *APOE* variants in a cohort of 229 Alzheimer patients and 89 healthy controls from the Iberian regions of Northern Portugal and Castile and León, Spain. We optimized a Sanger sequencing protocol to genotype rs429358 and rs7412 single nucleotide polymorphisms (SNPs) which define the APOE isoforms, overcoming technical difficulties usually experienced while assessing this GC-rich region [12]. Finally, we compared our *APOE* frequencies in AD cases and controls from the two regions with those reported for other Iberian and Caucasian populations.

## 2. Materials and Methods

### 2.1. Iberian Cohort

The cohort of this study was composed by a total of 229 clinically diagnosed Alzheimer patients with onset age ≥62 years old from Northern Portugal (*N* = 148) and Castile and León, Spain (*N* = 81), and 89 healthy controls ≥68 years old (*N* = 60 from Northern Portugal, and *N* = 29 from Castile and León). AD patients were clinically diagnosed following the criteria of the National Institute on Aging and Alzheimer’s Association (NIA-AA) [18] and with the exception of 6 subjects, all of them performed a Mini-Mental State Exam (MMSE) [19]. The main characteristics of studied individuals are shown in Table 1. Informed written consents were obtained from all the participants or from their family and/or legal representatives. This project has been approved by the Ethics Committee of the University of Porto (report #38/CEUP/2018), Portugal.

### 2.2. Sample Collection and DNA Extraction

Saliva and buccal swab samples were collected from patients and controls by using the saliva collector Oragene DNA OG-500 (DNA Genotek Inc., Ottawa, ON, Canada) or sterile brush/cotton buccal swabs. The choice of collection method was based on the ability of the patient to provide a 2 mL saliva sample with the specific saliva collector. In cases where this was not possible (often in patients in a severe/advanced state of the disease), sterile buccal swabs were used.

DNA was extracted according to the manufacturer’s instructions using the prepIT-L2P (DNA Genotek Inc., Ottawa, ON, Canada) for the saliva collectors and the Citogene Buccal Swab protocol from the Citogene Blood Kit (Citomed Lda., Odivelas, Portugal). For the buccal swab extraction procedure, an initial incubation step of 55 °C for 1–3 hours was introduced to ensure that most of dried epithelial cells were removed from the swab. After DNA extraction, samples were subjected to quantification of nucleic acids and quality control assessment by absorbance ratios (260/230 and 260/280) on a spectrophotometer (NanoDrop™ 1000, Thermo Fisher Scientific, Waltham, MA, USA). When necessary (i.e., poor Abs 260/230 ratio), a standard ethanol-based purification protocol was performed.

### 2.3. Whole Genome Amplification

Prior to the sequencing reaction, genetic material can be amplified to yield sufficient concentration of double-stranded DNA (dsDNA) in cases of low concentration [20] or simply to allow generation of more DNA for sample preservation (i.e., biobanking). To accomplish that aim, a genome-wide amplification protocol was performed according to the manufacturer’s instructions, using the Illustra GenomiPhi V2 DNA Amplification kit (GE Healthcare, Chicago, IL, USA). In summary, 9 μL of sample buffer was added to 1 μL of DNA (10 ng), and the mix was heated at 95 °C for 3 minutes before snap-cooling on ice. Subsequently, 9 μL of reaction buffer and 1 μL of enzyme were added to the mix, followed by an incubation at 30 °C for 1.5 hours and 65 °C for 10 minutes to inactivate the enzyme.

### 2.4. APOE Genotyping

Although next generation sequencing (NGS) techniques display numerous advantages, the first generation methodology Sanger sequencing [21] is still considered a solid and reliable method, especially in clinical settings. Sanger sequencing provides a way to confirm variants attributed by NGS, while also being able to cover regions poorly screened by those new technologies [22,23].

**Figure 1:**
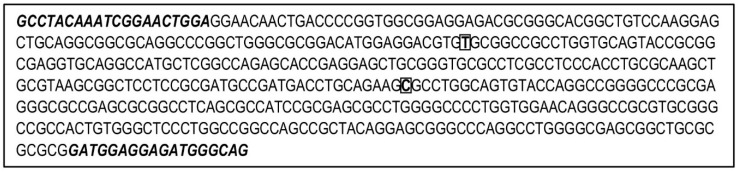
Sequenced region for *Apolipoprotein E* (*APOE*) including single nucleotide polymorphisms (SNPs) rs429358 (squared T in figure) and rs7412 (squared C in figure). Sequence downloaded from *UCSC Genome Browser* (https://genome.ucsc.edu) Assembly Dec. 2013, GRCh38/hg38, Chr19: 44908567-44909018. Designed forward and reverse primers for amplification and sequencing of target region are represented in italic and bold.

**Figure 2:**
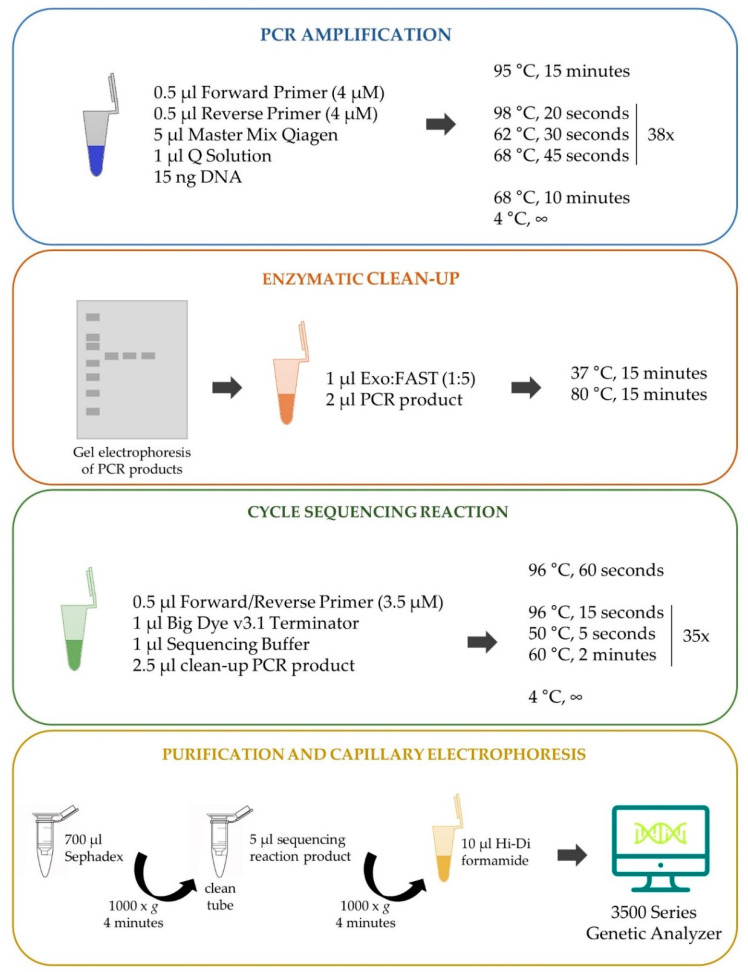
Schematic representation of the optimized protocol for *Apolipoprotein E* (*APOE*) genotyping.

To assess *APOE* variants, we sequenced a region encompassing both SNPs rs429358 (NC_000019.10:g.44908684T>C, GRCh38.p13) and rs7412 (NC_000019.10:g.44908822C>T, GRCh38.p13), after designing a single pair of primers (Primer3 tool; [24]) for amplification (Figure 1). Given the GC-rich DNA segment of our target region, a PCR amplification protocol was optimized as shown in Figure 2: 3 μL DNA (5 ng/μL) was added to a mix of 0.5 μL Forward Primer (4 μM) (5′-GCCTACAAATCGGAACTGGA, Merck SA, Algés, Portugal), 0.5 μL Reverse Primer (4 μM) (5′-CTGCCCATCTCCTCATC, Merck SA, Algés, Portugal), 5 μL master mix Qiagen, and 1 μL Q Solution (Qiagen^®^ Multiplex PCR kit, Qiagen, Hilden, Germany). The following thermocycling conditions were used, optimized from those reported by Kushioka et al. [12]: 95 °C for 15 minutes; 38 cycles of 98 °C for 20 seconds, 62 °C for 30 seconds, and 68 °C for 45 seconds; followed by a final extension step at 68 °C for 10 minutes, and cooling down to 4 °C. PCR efficacy was confirmed for all samples through a standard polyacrylamide gel 40% (w/v) acrylamide:bisacrylamide (19:1) solution and silver staining protocol. Next, we proceeded to an enzymatic clean-up step by adding 2 μL of the DNA product to 1 μL of 1:5 Exonuclease I (Thermo Fisher Scientific, Waltham, MA, USA) and FastAP Thermosensitive Alkaline Phosphatase (Thermo Fisher Scientific), followed by incubation at 37 °C and 80 °C, 15 minutes each. Samples were then submitted to cycle sequencing reaction (BigDye Terminator v3.1 Cycle Sequencing kit, Applied Biosystems, Foster City, CA, USA). The cleaned-up PCR product (2.5 μL) from the last step was added to the sequencing reaction mix containing the reverse or forward primer for sequencing (0.5 μL at 3.2 μM primer stock), 1 μL of Big Dye v3.1 Terminator, and 1 μL of Big Dye v3.1 Sequencing Buffer, totalizing a final 5 μL volume. The thermocycler conditions used were 96 °C for 1 minute; 35 cycles of 96 °C for 15 seconds, 50 °C for 5 seconds, and 60 °C for 2 minutes; followed by a final cool down step to 4 °C. A final purification step was performed with an Illustra™ Sephadex™ G-50 Fine DNA Grade solution (GE Healthcare), with 750 μL of Sephadex solution pipetted into columns inserted in 2 mL microcentrifuge tubes and centrifuged at 1000 × *g* for 4 minutes. Sephadex columns were then transferred to clean tubes and the total sequencing reaction product was carefully pipetted into the middle of the column before new centrifugation under the same conditions (1000 × *g*, 4 minutes). Finally, 10 μL of highly deionized formamide (Hi-Di™ Formamide, Applied Biosystems™) was added to each sample followed by capillary electrophoresis of the sequenced products (3500 Series Genetic Analyzer, Applied Biosystems, Thermo Fisher Scientific).

### 2.5. Sequencing Results and Statistical Analysis

The resulting sequencing data was analyzed using Unipro UGENE bioinformatic tool, v.33, available at www.ugene.net [25], with the designed primers of *APOE* SNPs for SNP localization, and the reference sequence GRCh38/hg38 for alignment.

Testing for deviations from Hardy–Weinberg equilibrium (HWE) was performed for both rs429358 and rs7412 SNPs in Portuguese and Spanish control populations, after Bonferroni correction, which resulted in a significance level α equal to 0.0125. To test for non-random distribution of haplotypes into population samples under the hypothesis of panmixia, we performed exact tests of differentiation (100,000 Markov steps; α = 0.05). Both HWE and exact tests were computed in Arlequin software [26].

To estimate odds ratios (ORs) of developing Alzheimer’s disease according to *APOE* genotypes and alleles, we used the online statistical software MedCalc^®^ (https://www.medcalc.org/calc/odds_ratio.php). ORs are presented with a 95% confidence interval (95% CI) using *APOE3/APOE3* genotype and *APOE3* allele as the reference in the analysis of *APOE* genotypes and alleles, respectively. When comparing ORs between males and females harboring the risk allele *APOE4*, we used *APOE4* non-carriers as the reference.

## 3. Results

### 3.1. Optimization of APOE Genotyping

Obtaining genotypes for *APOE* variants rs429358 and rs7412, which are responsible for APOE isoforms E2, E3, and E4, may present challenges due to amplification difficulties of the targeted fragment. The known difficulty in genotyping these two SNPs is most likely due to the high GC content of the region. In the present case, the amplicon flanked by the two primers used for both amplification and sequencing contains 74% of GC content (Figure 1).

Initial attempts to amplify and sequence this region using a standard amplification protocol were not successful, as we followed a basic PCR consisting of (1) an initial denaturation step at 95 °C, optimal temperature for our DNA polymerase activity; (2) 35 rounds of a three-step temperature cycle for denaturation at 94 °C, a fixed annealing temperature, and elongation at 68 °C; and (3) a final extension step at 68 °C. In a first attempt to solve the ineffective amplification, a touchdown PCR approach was used employing lower temperatures: a first annealing temperature above the projected Tm (Primer Melting Temperature), then transitioning to a lower, more permissive temperature over the course of 10–15 cycles [27]. Following this strategy, we tested an initial annealing temperature of 69 °C for 90 seconds transitioning to 60 °C after 10 cycles by a decrement of 1 °C per cycle. The second phase consisted of a generic amplification stage: 25 cycles at a Tm of 60 °C for 90 seconds. This approach did not improve the amplification results (in all PCR attempts both positive and negative controls were included and worked correctly). After deeper search in the literature, we applied the approach by Kushioka et al. [12], which considers a temperature of 98 °C for 10 seconds for denaturation (instead of a standard step at 94 °C) during the entire number of PCR cycles. We adapted this protocol to optimize our amplification reactions. Finally, we ended up with very strong and specific amplification bands by performing the key steps of denaturation at 98 °C for 20 seconds, and a Tm of 62 °C (Figure S1).

With the modification and optimization of a traditional *APOE* genotyping protocol, we expect to contribute to make more straightforward the assessment of variants that define APOE isoforms. Moreover, by describing a step-by-step protocol for genotyping such a GC-rich sequence, it is our aim to help others in the process of analyzing similar regions, not only for research purposes, but also in routine practices (Figure 2).

### 3.2. APOE Genetic Variation and LOAD ORs in Northern Portugal and in Castile and León Region, Spain

We tested conformity with the HWE expectations after Bonferroni correction for rs429358 and rs7412 in the Portuguese and Spanish control populations: no significant departures from equilibrium were found. Next, *APOE* allele and genotype frequencies were estimated for both patients and controls in each population group. Of the six possible *APOE* genotypes, only *APOE2/APOE4* was not observed in any of the cohorts (Table 2). Analyses of differentiation of allele and genotype distributions were computed within and between Portuguese and Spanish populations, for both cases and controls subgroups (Table 3). When analyzing cases versus controls, we found different allele and genotype distributions in both Portuguese and Spanish populations. We also found differences in allele frequencies between Portuguese and Spanish AD patients, which prevented us from pooling these populations in further genotype analyses.

To gain insight into the risk of developing LOAD as a function of the *APOE* alleles and genotypes in our Iberian populations, we estimated ORs for disease associated with *APOE* in our cohorts and compared to those calculated based on frequencies reported for a large Caucasian population [31] (Table 4). As expected, high ORs for LOAD were associated with *APOE4* alleles (2.17; CI, 1.19–3.99, in the Portuguese cohort; and 1.57; CI, 0.65–3.80, in the Spanish cohort). Moreover, given the importance of studying sex-risk factors for AD, we estimated *APOE4*-associated ORs for males and females from both Iberian populations (Table 5). Curiously, in the Portuguese cohort, our results have shown *APOE4* male carriers presenting slightly higher odds of developing LOAD (3.17; CI, 0.89–11.31) than *APOE4* female carriers (2.49; CI, 1.04–5.93); in the Spanish cohort, values 1.50; CI, 0.36–6.17 and 6.49; CI, 0.79–53.57 were observed, respectively. These results should, however, be interpreted with caution given the non-significance of most estimated ORs and the small sample size of some analyzed subgroups.

## 4. Discussion

In this study, we developed an optimized protocol for *APOE* genotyping by Sanger sequencing and described a step-by-step procedure to overcome the inherent drawbacks of the high GC content in *APOE*_exon 4, where SNPs rs429358 and rs7412 (that define *APOE* alleles) are located. Then, we estimated *APOE* genotypic and allelic frequencies in our cohorts of LOAD patients from Northern Portugal and Castile and León (Spain), in comparison to geographically matched control populations.

The three major *APOE* alleles display a worldwide frequency of approximately 8%, 78%, and 14%, respectively; nevertheless, a non-random global distribution has been reported in several studies [15,31,32,33,34]. In Europe, it has been observed a north-to-south gradient of *APOE4* and *APOE3* alleles, with frequency of *APOE4* increasing and *APOE3* allele frequency decreasing with latitude, whereas the observation of *APOE2* seems to be independent of the European latitude [34]. Interestingly, based on linkage disequilibrium evidence, Seixas et al. [29] suggested that the evolution of *APOE* alleles in humans followed a *E4*->*E3*->*E2* pathway, thus confirming *APOE4* as the ancestral allele. In their study, the authors reported *APOE* frequencies in the general population from Northern Portugal (*APOE2*: 4.4%; *APOE3*: 88.2%; and *APOE4*: 7.4%; Table 2). Recently, their work was cited in a comprehensive and elucidative review on *APOE* pleiotropy by Belloy et al. [15]; however, Portugal was placed distant from other European countries with a considerably deviating *APOE4* allele frequency. We herein take notice that frequencies reported in this review for the Portuguese population were mistakenly taken by those of the African population of São Tomé e Príncipe (*APOE2*: 10.0%; *APOE3*: 65.2%; and *APOE4*: 24.8%; Seixas et al., [29]). *APOE* frequencies in Portuguese and Spanish populations are rarely described in the literature, with a considerable gap on the comparison between AD patients and carefully selected controls. Our study was a prospective work with the aim of performing this comparison in the regions of Northern Portugal and Castile and León (Spain), to bridge the gap regarding Iberia and analyze whether or not *APOE* frequencies deviate from those reported for Caucasians.

*APOE2* alleles have been shown to confer a protective effect for AD. In addition to decreased amounts of AD-related brain pathology and a later disease age-of-onset in patients, *APOE2* variants are markedly underrepresented among affected individuals [35]. In our Portuguese subgroup, frequency of *APOE2* is reduced to about half in the cohort of patients when compared to controls (4.1% versus 7.5%; OR, 0.60; CI, 0.25–1.48), supporting the protective effect of these alleles. A similar reduction has been observed in a large meta-analysis comprising 5107 AD patients and 6262 controls from different Caucasian populations (OR, 0.61; CI, 0.54–0.69) (Table 2 and Table 4, [31]). On the other hand, frequency of *APOE2* in the general population from Northern Portugal and in a non-AD Portuguese cohort has been reported as low as 4.4% [29] and 6.3% [28], respectively. When retrieving *APOE* frequencies from other studies, we must take into account the aim of each study and also bear in mind that, even in case-control studies, *APOE* frequencies observed in control cohorts may include young age individuals at the time of examination, who may develop LOAD further in life; when this happens, frequencies described in such controls are biased by the presence of presymptomatic patients. In our study, we tried to overcome this issue by selecting controls aged ≥68 years old, free from any sign of dementia, submitted to the Mini-Mental State Exam (values ≥ 26). Interestingly, the presence of this protective allele has been detected in a single LOAD patient (heterozygous *APOE2*/*APOE3*) in our Spanish cohort, which resulted in a more accentuated decrease of *APOE2* in patients (0.6%) versus controls (8.6%), resulting in an OR of 0.07 (95% CI, 0.01–0.62); Table 4. This might be, however, explained by a random underrepresentation of rarer alleles, as a frequency of 6% has been previously reported for *APOE2* among LOAD patients from Madrid, Spain (even analyzing a small sample size of 47 patients; [30]). On the other hand, when looking at *APOE2* frequency among a geographically-matched control cohort, their results are not in line with a protective role ascribed for these alleles (6% in patients versus 5% in controls; Table 2). Again, the problem of including individuals examined at young age (controls ≥52 years old) can be raised, but in the study by Ibarreta et al. [30], the small sample size may also be underlying the difficult interpretation of results. Finally, the hypothesis that differences in detection methodologies may be causing some of these discrepancies cannot be discarded. To clarify whether such differences on *APOE2* allele frequencies do occur at regional level, it would be important to extend this study to other Spanish regions.

Our results for *APOE4* alleles—the strongest known genetic risk factor for LOAD—are similar to those previously reported in Caucasians [31]. In our Portuguese subgroup, the relative increase in *APOE4* frequency in patients is twice of that in controls (24.3% versus 12.5%; OR, 2.17; CI, 1.19–3.99), slightly below the reported for Caucasians (36.7% versus 13.7%; OR, 3.51; CI, 3.29–3.75); Table 3 and Table 4). In our Spanish subgroup, this increase is about 1.6-fold in AD patients (19.1%) when compared to controls (12.1%), very close to the ratio reported for Hispanic populations (19.2% versus 11%) [31]. Interestingly, in a study including subjects from the Spanish area of Madrid, a notorious increase of 8.5-fold in *APOE4* allele frequency in LOAD patients (34%) has been reported when compared to controls (4%) (Table 2, [30]). If, on one hand, *E4* frequency in Madrilenian patients is close to that reported among Caucasians (36.7%), the remarkable low frequency in controls is hard to explain looking at frequencies found either in Caucasians (13.7%), in our Spanish controls (12.1%) or among controls from the area of Barcelona (11.1%) (Table 2). The results should be interpreted with caution given the small sample size of the Madrid cohort (47 patients and 42 controls); nevertheless, it is very interesting to note such differences on *APOE* distribution at a regional level, highlighting the importance of these studies.

Globally, the most common isoform of the APOE protein is E3, believed to be neutral, not leading to an increased or decreased risk of developing AD. As expected, our results showed *APOE3* allele frequencies as the highest when compared to those of other variants, both in patients and controls from the two studied regions. Similar frequencies of *APOE3* in controls and patients are also in line with its neutral role in AD (Northern Portugal patients: 71.6%; controls: 80.0%; Castile and León patients: 80.2%; controls: 79.3%; Table 2).

In addition to the *APOE* genotype, sex-based differences in LOAD risk have been studied, but sex-dependent association of LOAD and *APOE* is still controversial and urges to be clarified. Several epidemiological studies have suggested that *APOE4* female carriers displayed a higher risk for LOAD when compared to their male counterparts [35,36,37], but complexity is added by studies showing that this finding may be additionally dependent on age [38,39]. For instances, in a large analysis comprising data on ~58,000 subjects from 27 independent studies in the Global Alzheimer’s Association Interactive Network, men and women carrying the *APOE4*/*APOE4* genotype had similar odds of developing AD across the age span of 55 to 85 years, with women showing an increased risk at younger ages [38]. In this study, we attempted to shed light into sex differences in *APOE4* effects on LOAD by comparing odds between males and females carrying the risk *APOE4* allele. We observed lower ORs in *APOE4* female carriers in the Portuguese cohort, however, the small sample size in some subgroups may be underlying this result and the non-significant ORs observed.

## 5. Conclusions

This work held the goal of studying the genetic variation of *APOE* in a cohort of late-onset AD patients from Northern Portugal and Castile and León (Spain), in comparison to geographically matched control populations. The genetic characterization of *APOE* provides information on the landscape of AD in these regions based on the haplotype data obtained from *APOE* alleles at SNPs rs429358 and rs7412. We developed an optimized protocol for *APOE* genotyping through Sanger sequencing, overcoming the inherent drawbacks of high GC content in this genomic region. Our results on *APOE* frequencies are in line with previous reports, although differences regarding the rare *APOE2* allele may be further explored by extending this study to other Iberian subgroups. Moreover, discrepancies on *APOE4* allele frequencies reported in other studies of Portuguese and Spanish cohorts deserve a closer look. Finally, we have drawn attention to the importance of selecting carefully control individuals in AD studies given the late onset of this disease. By capturing a broader and accurate picture of *APOE* allelic variation in multiple populations (including at regional levels), we hope to contribute to a comprehensive evaluation of the utility of *APOE* in a clinical context.

## Figures and Tables

**Table 1:** Characteristics of analyzed late onset Alzheimer’s disease (LOAD) patients and controls from Northern Portugal (PT) and Castile and León Spanish region (SP).

		Controls		LOAD Patients					
				MIL		MOD		SEV	
Population		PT	SP	PT	SP	PT	SP	PT	SP
**Females (*N*)**		37	13	20	14	27	21	66	22
**Males (*N*)**		23	16	7	14	8	5	20	5
**Examination age (years)**	**Range**	70–96	68–87	65–93 *	62–89	67–96	63–92	65–99	66–94
	**Mean ± SD**	84.3 ± 7.3	77.3 ± 4.8	80.2 ± 7.6	78.1 ± 6.9	83.4 ± 8.1	79.9 ± 7.6	83.4 ± 7.8	82.8 ± 7.0
**MMSE**	**Range**	26–30	27–30	12–24 **	20–30	5–28 **	11–20	0–10	0–10
	**Mean ± SD**	28.2 ± 1.2	29.1 ± 1.1	20.0 ± 3.6	23.2 ± 2.5	12.0 ± 4.6	14.7 ± 2.7	1.3 ± 2.9	4.4 ± 3.9

* Information about age not available for 1 individual. ** MMSE scores not available for 3 individuals. MIL: patients with mild Alzheimer’s disease; MOD: patients with moderate Alzheimer’s disease; SEV: patients with severe Alzheimer’s disease. *N*: Number of individuals.

**Table 2:** Allele and genotype frequencies of *Apolipoprotein E* (*APOE*) variants in late onset Alzheimer’s disease (LOAD) patients and controls observed in our cohorts from Northern Portugal and Castile and León Spanish region in comparison to those reported in the literature. *N*: number of individuals; NA: not available.

Status	Population	Source	*N*	*APOE* Allele Frequency (%)			*APOE* Genotype Frequency (%)					
				*E2*	*E3*	*E4*	*E2/E2*	*E2/E3*	*E2/E4*	*E3/E3*	*E3/E4*	*E4/E4*
**Controls**	**Portuguese**	This study	60	7.5	80.0	12.5	1.7	11.7	0	66.7	15.0	5.0
	**Portuguese**	Haddy et al., 2002 [28]	607 ^§^	6.3	84.3	9.4	0.3	11.0	1.0	71.2	15.2	1.3
	**Portuguese**	Seixas et al., 1999 [29]	149 ^ʄ^	4.4	88.2	7.4	0	8.7	0	77.9	12.1	1.3
	**Spanish**	This study	29	8.6	79.3	12.1	0	17.2	0	65.5	10.3	6.9
	**Spanish**	Ibarreta et al., 1995 [30]	42 ^#^	5.0	92.0	4.0	NA	NA	NA	NA	NA	NA
	**Spanish**	Haddy et al., 2002 [28]	906 ^§§^	7.8	81.1	11.1	0.9	12.7	1.1	64.9	19.6	0.8
	Caucasian ***	Farrer et al., 1997 [31]	6262 *	8.4	77.9	13.7	0.8	12.7	2.6	60.9	21.3	1.8
**Patients**	**Portuguese**	This study	148	4.1	71.6	24.3	1.4	5.4	0	52.7	32.4	8.1
	**Spanish**	This study	81	0.6	80.2	19.1	0	1.2	0	64.2	30.9	3.7
	**Spanish**	Ibarreta et al., 1995 [30]	47^##^	6.0	60.0	34.0	NA	NA	NA	NA	NA	NA
	Caucasian ***	Farrer et al., 1997 [31]	5107 **	3.9	59.4	36.7	0.2	4.8	2.6	36.4	41.1	14.8

^§^ Individuals aged 25–64 years, free from serious and/or chronic illnesses (including AD) at the time of the recruitment, who attended different laboratories of the National Institute of Health of Lisbon. ^§§^ Individuals aged 25–64 years, free from serious and/or chronic illnesses (including AD) at the time of the recruitment, from the area of Barcelona. ^ʄ^ Unrelated individuals born and living in Northern Portugal; information on age of individuals not available. ^#^ Individuals aged 52–90 years (mean ± SD, 67.7 ± 1.9) without any clinical symptom of AD from the area of Madrid. ^##^ LOAD patients diagnosed in the Department of Neurology of the ‘Hospital Universitario Doce de Octubre’ (Madrid, Spain). * Controls aged between (mean ± SD) 37.2 ± 10.6 and 82.5 ± 8.4, free of neurodegenerative and neuropsychiatric illnesses. ** Patients diagnosed as having definite or probable AD excluding those with known mutations in the *APP* or presenilin genes or coexisting neuropathological findings (e.g., Lewy bodies, Parkinson disease changes). *** Subjects categorized as Caucasians by Farrer et al. [31], from the analysis of 37 different studies including AD patients and controls designated as: Caucasian (6264), Anglo-Saxon (667), French (704), French Canadian (792), Scandinavian (77), Finnish (259), Italian (280), Dutch (1848), German (270), Ashkenazi Jewish (201), and Sephardic Jewish (5).

**Table 3:** *Apolipoprotein E* (*APOE*) allelic and genotypic differences of late onset Alzheimer’s disease (LOAD) patients versus controls for Portuguese and Spanish cohorts. For allele distribution, we also tested differences of Portuguese versus Spanish for cases and controls. Significant *p*-values ± SD (α = 0.05) obtained from exact tests of differentiation are marked with an asterisk. After finding differences on *APOE* allele frequencies between Portuguese and Spanish patients (*P* = 0.03265), we have not performed further interpopulation analyses (marked in the table as NC: not calculated). *N*: Number of individuals or alleles when *APOE* genotypes or *APOE* alleles are analyzed, respectively.

		Portugal		Spain	
		LOAD Patients	Controls	LOAD Patients	Controls
		**Allele Distribution**			
**Portugal**	**LOAD patients** **(*N* = 296)**		0.00887 ± 0.00173 *	0.03265 ± 0.00504 *	NC
	**Controls** **(*N* = 120)**			NC	0.95815 ± 0.00102
**Spain**	**LOAD patients** **(*N* = 162)**				0.00560 ± 0.00118 *
	**Controls** **(*N* = 58)**				
		**Genotype Distribution**			
**Portugal**	**LOAD patients** **(*N* = 148)**		0.03801 ± 0.00308 *	NC	NC
**Spain**	**LOAD patients** **(*N* = 81)**	NC	NC		0.00232 ± 0.00051 *

**Table 4:** Odds ratios of developing late onset Alzheimer’s disease according to *Apolipoprotein E* (*APOE*) genotypes and alleles among subjects from our Portuguese and Spanish cohorts in comparison to those calculated from reported *APOE* frequencies in a meta-analysis including several Caucasian populations (Farrer et al., 1997; [31]). *N*: Number of individuals or alleles when *APOE* genotypes or *APOE* alleles are analyzed, respectively.

Population	*APOE* Genotype/Allele	Controls (*N*)	Patients (*N*)	Odds Ratio (95% Confidence Interval)	*p*-Value
Portuguese (this study)	*E3E3* (Referent)	40	78	1	-
	*E2E2*	1	2	1.03 (0.09 to 11.66)	0.9837
	*E2E3*	7	8	0.59 (0.20 to 1.73)	0.3338
	*E2E4*	0	0	-	-
	*E3E4*	9	48	2.74 (1.22 to 6.13)	0.0146
	*E4E4*	3	12	2.05 (0.55 to 7.69)	0.2865
	*E3* (Referent)	96	212	1	-
	*E2*	9	12	0.60 (0.25 to 1.48)	0.2704
	*E4*	15	72	2.17 (1.19 to 3.99)	0.0121
Spanish (this study)	*E3E3* (Referent)	19	52	1	-
	*E2E2*	0	0	-	-
	*E2E3*	5	1	0.07 (0.01 to 0.67)	0.0203
	*E2E4*	0	0	-	-
	*E3E4*	3	25	3.04 (0.82 to 11.26)	0.0952
	*E4E4*	2	3	0.55 (0.08 to 3.54)	0.5274
	*E3* (Referent)	46	130	1	-
	*E2*	5	1	0.07 (0.01 to 0.62)	0.0169
	*E4*	7	31	1.57 (0.65 to 3.80)	0.3206
Caucasian (Farrer et al., 1997)	*E3E3* (Referent)	3813	1859	1	-
	*E2E2*	50	10	0.41 (0.21 to 0.81)	0.0104
	*E2E3*	795	245	0.63 (0.54 to 0.74)	<0.0001
	*E2E4*	163	133	1.67 (1.32 to 2.12)	<0.0001
	*E3E4*	1328	2104	3.25 (2.97 to 3.55)	<0.0001
	*E4E4*	113	756	13.72 (11.18 to 16.85)	<0.0001
	*E3* (Referent)	9756	6067	1	-
	*E2*	1052	398	0.61 (0.54 to 0.69)	<0.0001
	*E4*	1716	3749	3.51 (3.29 to 3.75)	<0.0001

**Table 5:** Odds ratios of developing late onset Alzheimer’s disease for *Apolipoprotein E4* (*APOE4*) carriers from our analyzed Portuguese and Spanish populations. *N*: Number of individuals.

		Portuguese Cohort			Spanish Cohort		
		Total	Females	Males	Total	Females	Males
Patients (*N*)	*APOE4* carriers	60	46	14	28	20	8
	*APOE4* non-carriers	88	67	21	53	37	16
Controls (*N*)	*APOE4* carriers	12	8	4	5	1	4
	*APOE4* non-carriers	48	29	19	24	12	12
Odds ratio (95% confidence interval)		2.73 (1.34 to 5.56)	2.49 (1.04 to 5.93)	3.17 (0.89 to 11.31)	2.54 (0.87 to 7.37)	6.49 (0.79 to 53.57)	1.50 (0.36 to 6.17)
*p*-value		0.0058	0.0395	0.0759	0.0873	0.0826	0.5742

## Supplementary Materials

The following are available online at https://www.mdpi.com/2073-4425/12/1/4/s1, Figure S1: Amplification products resulted from the optimized protocol for *Apolipoprotein E* (*APOE*) genotyping. M: Molecular-weight size marker (with respective sizes in base pairs, bp); NC: Negative Control; 1: sample 1; 2: sample 2.
