# Supplementary figures and images for: APOE Variants in an Iberian Alzheimer Cohort Detected through an Optimized Sanger Sequencing Protocol
Source: Genes (Basel). 2020 Dec 22;12(1):4. doi: 10.3390/genes12010004 (PMC7822120; doi:10.3390/genes12010004)

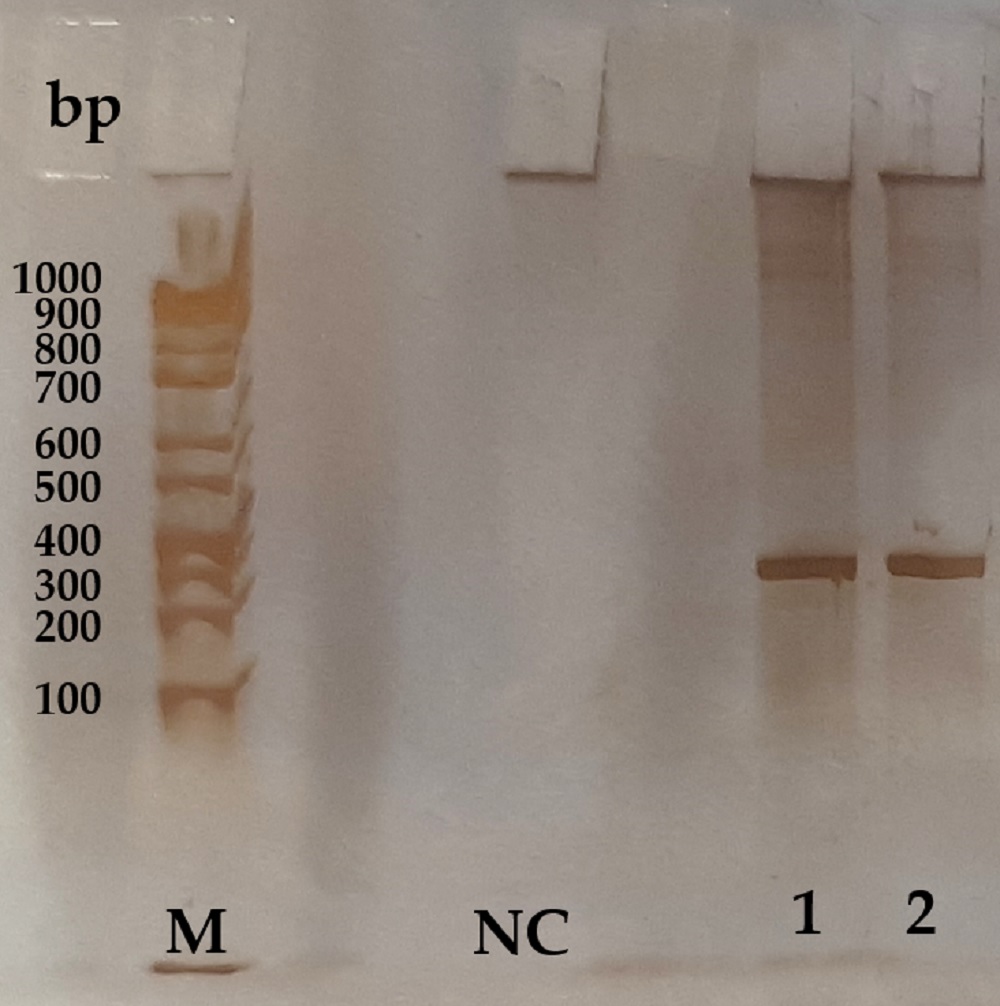

Supplement: Supplementary file 1 [file genes-12-00004-s001.zip › genes-1020495-supplementary.jpg]
